# Clinical Features and Prognostic Analysis of Elderly Patients With Late‐Onset Epilepsy

**DOI:** 10.1002/brb3.70452

**Published:** 2025-04-21

**Authors:** Jie Hu, Long Wang, Jun‐Cang Wu

**Affiliations:** ^1^ Department of Neurology Hefei Hospital Affiliated to Anhui Medical University (The Second People's Hospital of Hefei) Hefei Anhui China; ^2^ Department of Neurology The Fifth Clinical Medical College of Anhui Medical University Hefei Anhui China

**Keywords:** Comorbidities, Elderly patients, Late‐onset epilepsy, Prognosis, Seizure control

## Abstract

**Purpose:**

To analysis the basic characteristics, comorbidities and prognosis of elderly patients with Late‐Onset Epilepsy (LOE) in the Eastern Region of Hefei, Anhui.

**Methods:**

This study finally selected 304 participants who were enrolled at the Second People's Hospital of Hefei between January 2018 and December 2023. The analysis included baseline characteristics, etiology, seizure types, findings from electroencephalography (EEG) and cranial magnetic resonance imaging, comorbidities, anti‐seizure medication (ASM) regimens, and follow‐up of seizure control outcomes within one year. Continuous variables were presented as mean ± standard deviation (SD) or median (IQR) based on normality. Categorical variables were compared using the chi‐square test with Bonferroni correction for multiple comparisons.

**Results:**

According to our study, ischemic cerebral infarction (41.12%) was the main factor for LOE in elderly patients among structural factors. Focal seizure (92.76%) was the main seizure type. The most common comorbidity was ischemic cerebral infarction (88.16%), followed by cerebral hemorrhage (22.37%). During the one year follow‐up, the overall effectiveness of seizure control was 73.03%, and 49.34% patients were seizure‐free. The one‐year treatment efficacy of patients with comorbid psychiatric disorders, cognitive impairment or dementia were significantly lower than that of patients without these comorbidities. In terms of medications, sodium valproate accounted for the most at 86.84%.

**Conclusion:**

Structural factors are the main etiology for LOE in elderly patients, with ischemic cerebral infarction accounting for the highest proportion. Focal seizure was the main seizure type. Patients with comorbid psychiatric disorders, cognitive impairment or dementia may have poor one‐year treatment efficacy.

## Graphical abstract

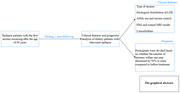

**Abbreviations**
ASM: anti‐seizure medication
CVD: cerebrovascular disease
EEG: electroencephalography
FDA: Food and Drug Administration.
FHS: Framingham Heart Study
ICD: International Classification of Diseases
ILAE: International League Against Epilepsy
IQR: interquartile range
LOE: late‐onset epilepsy
MRI: magnetic resonance imaging
PSE: post‐stroke epilepsy
QoL: quality of life
SD: standard deviation

## 1 Introduction

As a common neurological disorder, epidemiologic studies have shown that the prevalence and incidence of epilepsy are usually age‐related, with a significantly higher incidence of epilepsy in the elderly population than in other age populations (Fiest et al. 2017). The proportion of people over 65 years of age is growing globally with the increasement in population aging. In some industrialized countries, one‐third of the population is estimated to exceed 65 years by 2023 (Stefan 2011). Therefore, we are facing a growing population of older adults with epilepsy. Epilepsy is one of the most common neurological disorders in older adults, secondly to stroke and dementia, and because of transient loss of consciousness from various causes becomes common with advancing age, which made diagnosis and differentiation of epilepsy difficult (Werhahn 2009). In addition, elderly with epilepsy may have different characteristics in terms of etiology, clinical features, and disease control (Brodie et al. 2009), posing a serious challenge to the healthcare system. However, relatively little research has been conducted on this population compared to younger individuals.

Comorbidities are defined as concomitant diseases in the development of a given disease (Feinstein 1970). In the elderly population, the comorbidities of epilepsy are diverse and extensive. Previous studies have shown that epilepsy may be caused by an abnormal tendency due to a combination of one or more disorders. Therefore, any comorbidity should be considered as one of the directions that should be taken into account in the epilepsy treatment program (Engel 2006, Keezer et al. 2016). Recurrent LOE in elderly has a great impact on the patient's quality of life in their later years, and each uncontrolled seizure can seriously post a heavy burden on the patients. Hence, there is an urgent need for more in‐depth study on the key clinical features and prognosis of elderly patients with LOE. Moreover, since the risk of new epileptic seizures is high in people older than 60 years old, in this study, we conducted a study to analysis elderly patients with LOE, which is defined with the first seizure occurring at the age of 60 years or older (Johnson et al. 2018, Vu et al. 2018), so as to provide a clear reference for disease control in this group of patients.

## 2 Materials and methods

### 2.1 Patient Selection and Follow‐up

Participants of the Second People's Hospital of Hefei between January 2018 and December 2023 were consecutively selected through the electronic medical record (EMR) system. All patients met the epilepsy diagnostic criteria established by the International League Against Epilepsy (ILAE) 2017 and underwent electroencephalography (EEG) assessments to confirm the diagnosis. The follow‐up period began on the date when the patient confirmed the diagnosis, completed the medical history collection and relevant examinations, with the endpoint defined as one year thereafter. Follow‐up data were obtained from EMR and telephone interviews to ensure data completeness. The inclusion criteria were: 1. Patients with the first epilepsy code assigned after the age of 60 years (screened according to the International Classification of Diseases (ICD) ‐10 codes) and no history of ASM use; 2. Complete medical history with EEG, MRI, and other findings within 24 to 72 hours of inclusion in the study; 3. Completed one year of follow‐up after enrolled from the hospital. 4. Patients who experienced a single seizure but had EEG or MRI evidence supporting an epilepsy diagnosis. Exclusion criteria were as followed: 1. Missing clinical information; 2. Loss to follow‐up or death during one year of follow‐up; 3. The first epilepsy code assigned before the age of 60 years; 4. Patients with a single unprovoked seizure or acute symptomatic seizures.

### 2.2 Medical History and Data Collection

Clinical information was collected through the EMR system, including baseline characteristics (age, sex, alcohol and smoking status), seizure type and etiology, comorbidities, therapeutic approach, 3.0T cranial magnetic resonance imaging (MRI) and electroencephalography (EEG) results. According to the ILAE 2017 epilepsy classification criteria, seizures were categorized as focal, generalized and unclassifiable origins, and etiology was categorized as structural, hereditary, infectious, immunologic, metabolic and unknown (Scheffer et al. 2017). The comorbidities include ischemic stroke, cerebral hemorrhage, intracranial space‐occupying lesions, central nervous system infections and immune disorders, traumatic brain injury, dementia, and systemic vascular risk factors (e.g., hypertension, diabetes mellitus and coronary artery disease, whose association with epilepsy was inconsistent in different studies (Johnson et al. 2018, Pugh et al. 2009)). Seizure‐free or a reduction in seizure frequency of more than 50% was classified as effective treatment with ASMs (ASMs effective), whereas a reduction of less than 50% or an increase in seizure frequency was considered ineffective treatment with ASMs (ASMs non‐effective) at follow‐up (Huang et al. 2016).

### 2.3 Ethics and Statement

The research protocol was reviewed and approved by the Ethics Committee of Hefei Hospital Affiliated to Anhui Medical University (Ethical Approval Number: 2023‐SR‐006‐01). Written informed consent was obtained from all participants before enrollment, allowing the use and publication of their clinical data. All data were analyzed anonymously to ensure confidentiality and compliance with ethical standards.

### 2.4 Statistical Methods

To assess data normality, the Kolmogorov‐Smirnov test was used, while the Levene test was applied to evaluate variance homogeneity. Normally distributed continuous variables were presented as mean ± standard deviation (SD), whereas non‐normally distributed variables were expressed as median (interquartile range, IQR). Categorical variables were compared using the chi‐square test with Bonferroni correction for multiple comparisons. A two‐tailed p‐value of less than 0.05 indicated a statistically significant difference. Specific statistical analyses were performed by using SPSS 25.00.

## 3 Results

**FIGURE 1:**
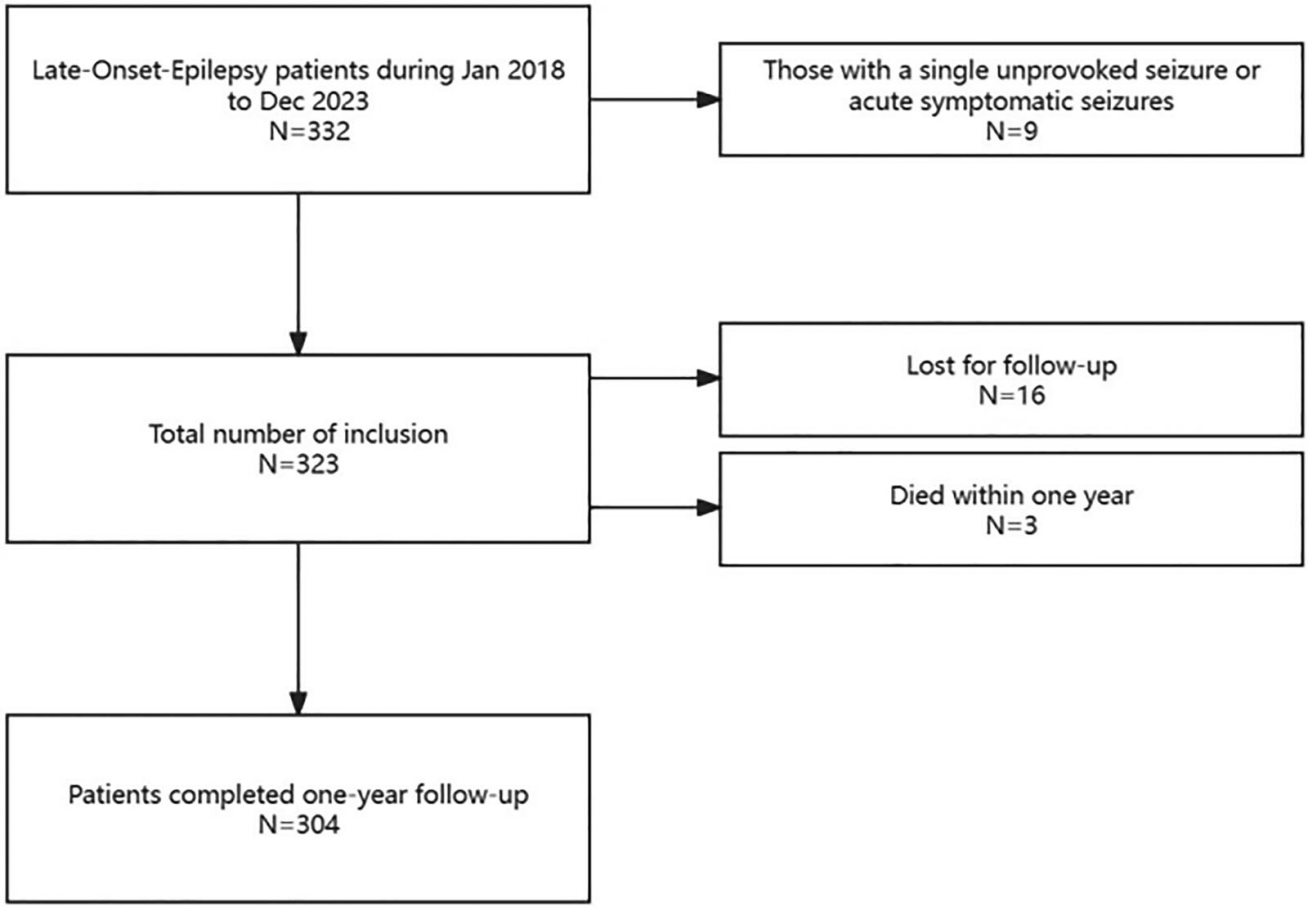
The flowchart of patients recruitment and follow‐up.

A total of 332 patients who met the criteria were included, and 9 were excluded since they only had 1 single unprovoked seizure. During the one‐year follow‐up period, 16 patients were lost to follow‐up, 3 patients died (Two patients died due to brain herniation following neurological intervention for massive cerebral infarction. One patient, who had acute cerebral infarction complicated by uncontrolled epilepsy, failed to receive further treatment. The patient's death was confirmed during a telephone follow‐up), and finally selected 304 patients, more details can be seen in Figure 1.

The mean age of the patients was 74.01 ± 7.72 years, and the percentage of males was 61.51%. According to Table 1, the etiological distribution, cognitive impairment or dementia, neoplasms and psychiatric disorders differed significantly between the two groups (P< 0.05).

**TABLE 1:** Baseline characteristics and clinical data of 304 patients with late‐onset epilepsy.

Characteristics	Total (n = 304)	ASMs non‐effective (n = 82)	ASMs effective (n = 222)	*χ^2^/t*	*P*
Age, Mean ± SD	74.01 ± 7.72	73.55 ± 8.26	74.18 ± 7.53	t=‐0.64	0.525
Sex, n(%)				χ^2^=1.39	0.238
Female	117 (38.49)	36 (43.90)	81 (36.49)		
Male	187 (61.51)	46 (56.10)	141 (63.51)		
Smoking, n(%)				χ^2^=0.12	0.733
No	249 (82.72)	68 (83.95)	181 (82.27)		
Yes	52 (17.28)	13 (16.05)	39 (17.73)		
Alcohol comsumption, n(%)				χ^2^=0.15	0.695
No	260 (86.38)	71 (87.65)	189 (85.91)		
Yes	41 (13.62)	10 (12.35)	31 (14.09)		
Etiology, n(%)				—	0.003
Immune	4 (1.32)	2 (2.44)	2 (0.90)		
Infectious	1 (0.33)	0 (0.00)	1 (0.45)		
Metabolic	13 (4.28)	5 (6.10)	8 (3.60)		
Structural	184 (60.53)	60 (73.17)	124 (55.86)		
Genetic	0	0	0		
Unknown	102 (33.55)	15 (18.29)	87 (39.19)		
MRI, n(%)					
Lesion location					
Unilateral	147 (72.77)	40 (80.00)	107 (71.81)	χ^2^=0.15	0.607
Bilateral	55 (27.23)	13 (20.00)	42 (28.19)		
Lesions accumulate in brain					
One Brain Lobe	103	25	78	χ^2^=0.12	0.517
Two or More Brain Lobes	99	28	71		
Seizure type, n(%)				—	1.000
Focal seizure	282 (92.76)	76 (92.68)	206 (92.79)		
Generalized seizure	15 (4.93)	4 (4.88)	11 (4.95)		
Unclassifiable	7 (2.30)	2 (2.44)	5 (2.25)		
EEG, n(%)				χ^2^=6.86	0.077
Epileptiform discharge	36 (11.84)	7 (8.54)	29 (13.06)		
Focal	57 (18.75)	9 (10.98)	48 (21.62)		
Generalized	88 (28.95)	29 (35.37)	59 (26.58)		
Normal	123 (40.46)	37 (45.12)	86 (38.74)		
Comorbidities, n(%)					
Ischemic stroke, n(%)				χ^2^=1.73	0.188
No	36 (11.84)	13 (15.85)	23 (10.36)		
Yes	268 (88.16)	69 (84.15)	199 (89.64)		
Hemorrhagic stroke, n(%)				χ^2^=1.29	0.257
No	236 (77.63)	60 (73.17)	176 (79.28)		
Yes	68 (22.37)	22 (26.83)	46 (20.72)		
Neoplasms, n(%)				χ^2^=6.11	0.013
No	279 (91.78)	70 (85.37)	209 (94.14)		
Yes	25 (8.22)	12 (14.63)	13 (5.86)		
Dementia or cognitive impairment, n(%)				χ^2^=12.42	< 0.001
No	272 (89.47)	65 (79.27)	207 (93.24)		
Yes	32 (10.53)	17 (20.73)	15 (6.76)		
Psychiatric disorders, n(%)				χ^2^=64.39	< 0.001
No	229 (75.33)	35 (42.68)	194 (87.39)		
Yes	75 (24.67)	47 (57.32)	28 (12.61)		
Vascular risk factors, n(%)					
Hypertension, n(%)				χ^2^=0.02	0.878
No	91 (29.93)	24 (29.27)	67 (30.18)		
Yes	213 (70.07)	58 (70.73)	155 (69.82)		
Diabetes, n(%)				χ^2^=0.25	0.618
No	225 (74.01)	59 (71.95)	166 (74.77)		
Yes	79 (25.99)	23 (28.05)	56 (25.23)		
Coronary artery disease, n(%)				χ^2^=0.00	0.960
No	259 (85.20)	70 (85.37)	189 (85.14)		
Yes	45 (14.80)	12 (14.63)	33 (14.86)		
Therapeutic approach, n(%)				—	0.820
No medication	17 (5.59)	3 (3.66)	14 (6.31)		
Monotherapy	217 (71.38)	59 (71.95)	158 (71.17)		
Two AEDs	57 (18.75)	17 (20.73)	40 (18.02)		
Three or more AEDs	13 (4.28)	3 (3.66)	10 (4.50)		

Type of seizure: The focal seizure was the most common type (92.76%), and generalized seizures were rare (4.93%).

**FIGURE 2:**
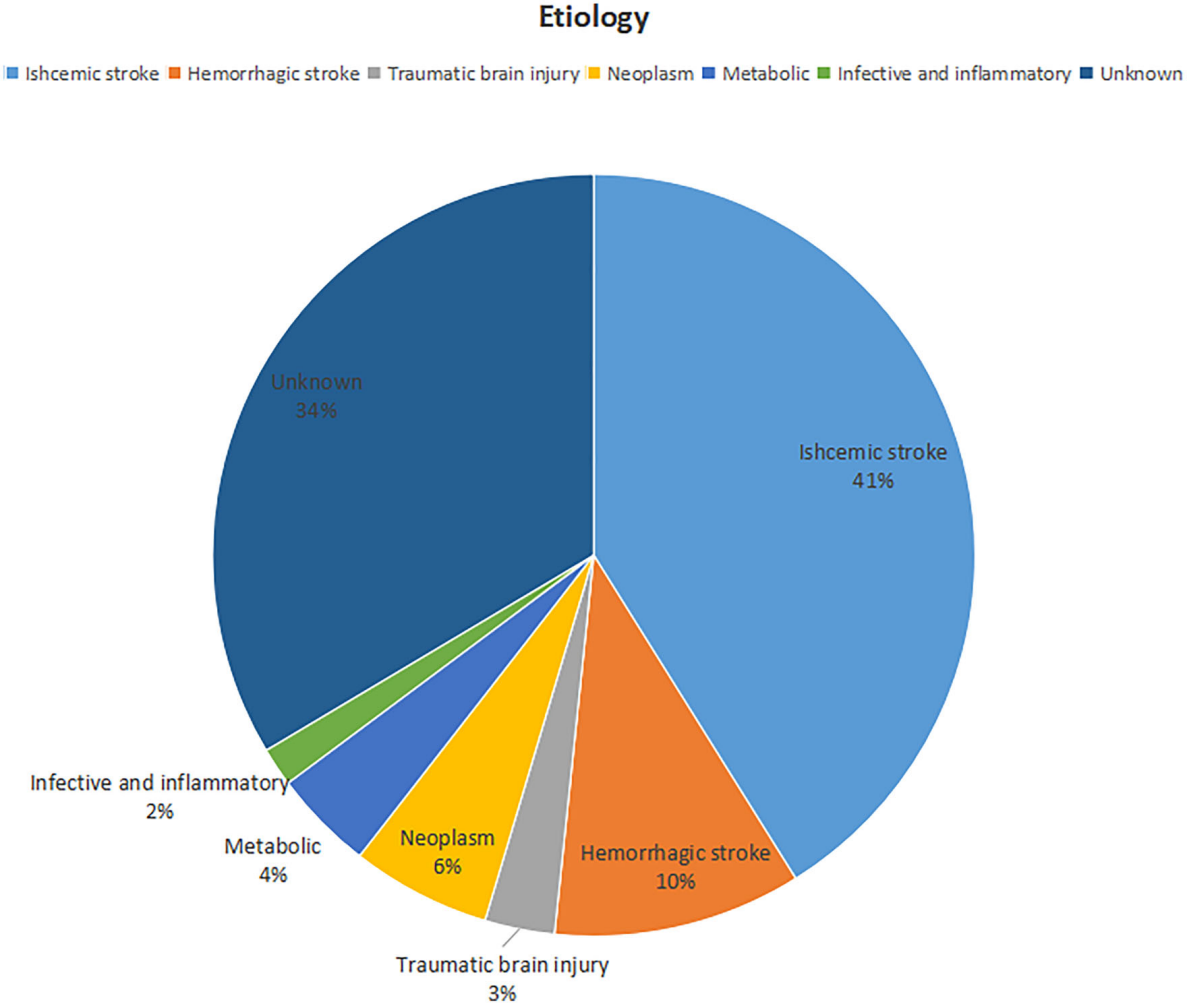
Etiological distribution of late‐onset epilepsy.

Etiological distribution of LOE: The results of this study show that ischemic stroke (41.12%), a structural factor, was the most common etiology in elderly patients with LOE. The second most common etiologies were cerebral hemorrhage (10.53%) and intracranial space‐occupying lesions (5.92%) (Figure 2).

ASMs use and seizure control: Monotherapy was the most commonly used ASMs regimen, accounting for 71.38% of the cases, while the number of ASMs used did not significantly correlate with the effectiveness of seizure control. A total of 264 patients used ASMs, including sodium valproate in 86.84% of patients and phenobarbital in 13.16% of patients. During the 1 year, A total of 222 patients experienced a significant reduction in seizure frequency and were classified into the ASMs effective group(222/304, 73.03%). A total of 179 patients were seizure‐free(150/304, 49.34%), while 82 patients experienced a seizure frequency reduction of less than 50% or a increase in seizure frequency, these 82 patients were classified into the ASMs non‐effective group(82/304, 26.97%).

EEG and cranial MRI: A total of 202 participants completed the cranial MRI, which revealing that unilateral or single‐lobe brain lesions were more prevalent. Epileptogenic lesions were confined to unilateral side in 72.77% of patients, while 50.99% exhibited a single epileptogenic lesion. All patients completed EEG examinations, and 59.54% showed EEG abnormalities.

**FIGURE 3:**
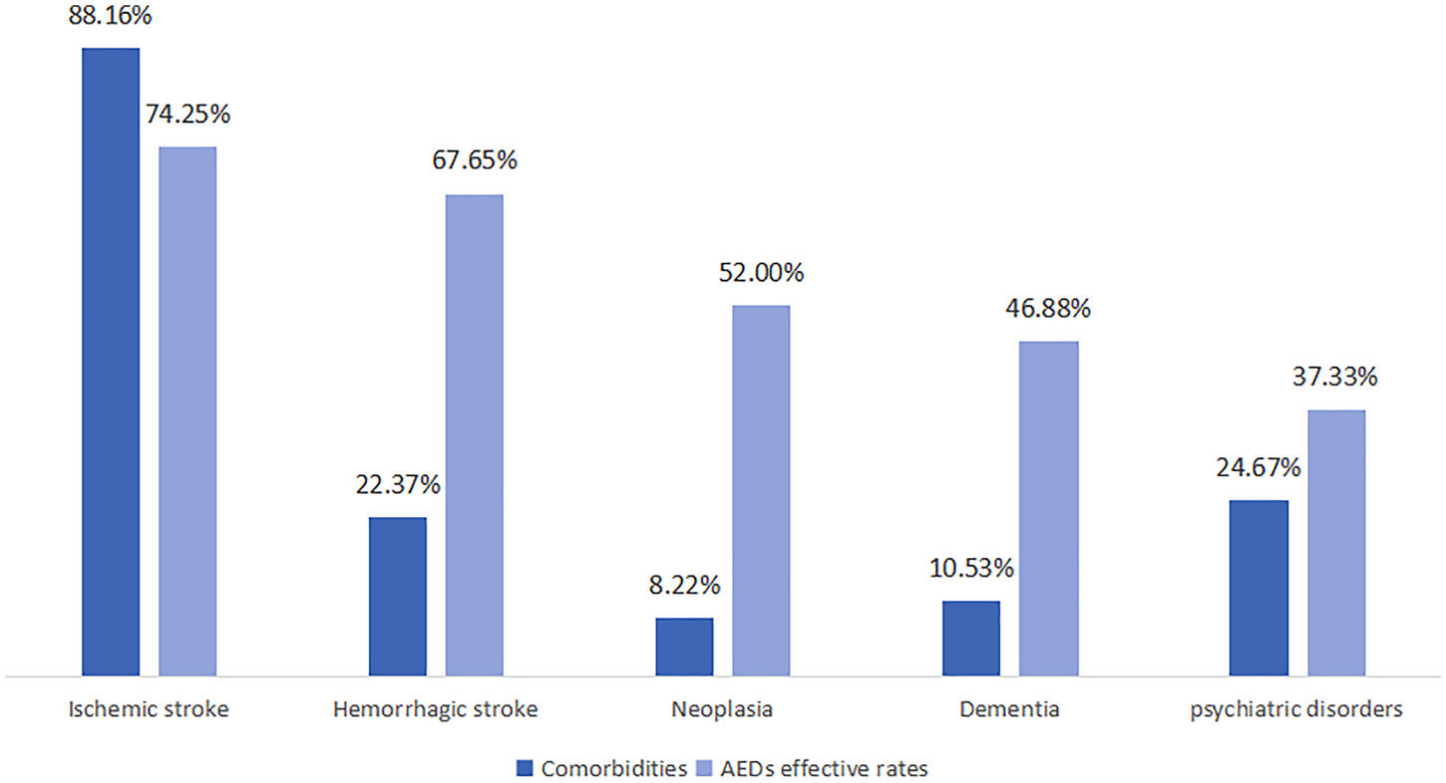
Comorbidities distribution and percentage of effective control of ASMs.

Comorbidities: A total of 88.16% of patients had cerebral infarction. The prevalence of cerebral hemorrhage, intracranial space‐occupying lesions or neoplasms, and psychiatric disorders was 22.37%, 8.22%, and 24.67%, respectively. Cognitive impairment or dementia was observed in 10.53% of participants. Additionally, hypertension, diabetes mellitus, and coronary artery disease were present in 70.07%, 25.99%, and 14.80% of participants, respectively (Figure 3).

## 4 Discussion

This study found that the most common type of seizure in elderly patients with LOE was focal seizure, while generalized seizures were less common, which is in line with previous studies (Huang et al. 2016). And we concluded that high prevalence of structural lesions was a major contributing factor to the high proportion of focal seizures, with most cases of post‐stroke epilepsy (PSE) resulting from focal brain lesions. Focal seizures usually involve neuronal networks within a localized region of brain tissue, whereas generalized seizures typically result from synchronized discharges across multiple, widely distributed foci. The MRI findings in this study also indicated that unilateral or a single‐lobe lesion were more commonly observed; In 72.77% of patients, epileptogenic lesions were confined to one hemisphere, and 50.99% had a single epileptogenic lesion on MRI. The type of seizure corresponded to the location of the epileptogenic lesion, which further validating our results. However, some previous studies have found that generalized seizures are the most common type in elderly patients (Tabatabaei et al. 2013), accounting for 75% of the cases. A possible explanation for this discrepancy may be the differences in the studied populations' ethnicity and lifestyle. In addition, some studies have suggested that the main reason for this discrepancy may be attributed to the higher prevalence of brain atrophy and white matter lesions in the elderly. An aging brain may be less capable of restricting the spread of epileptic activity, finally leading to a higher incidence of generalized seizures (Kellinghaus et al. 2004).

The results of this study were consistent with previous findings that ischemic stroke (41.12%), a structural factor, was the most common etiology in elderly patients with LOE. The second most common etiologies were cerebral hemorrhage (10.53%) and intracranial space‐occupying lesions (5.92%), differing from a previous study that concluded ischemic stroke was the most common etiology, followed by traumatic brain injury and intracranial space‐occupying lesions (Arabi et al. 2018). The target population screened in this study was neurological patients. Nonetheless, patients with intracranial space‐occupying lesions or traumatic brain injury were mostly triaged to neurosurgery or oncology for further treatment, the difference in the percentage was negligible.

Post‐stroke epilepsy (PSE) is the most common cause of seizures in older adults. Current research suggests that multiple factors contribute to its development, including gliosis, deafferentation, selective neuronal loss, chronic inflammation, angiogenesis, neurodegeneration, and changes in synaptic plasticity. Ultimately, these pathways create a hyperexcitable state, contributing to the development of PSE (Altman et al. 2019). The effectiveness of seizure control in patients with ischemic cerebral infarction as the etiology was 70.4%, and previous studies have also demonstrated a better prognosis of epilepsy after stroke (Feyissa et al. 2019). However, the potential mechanisms remain unclear, one possible explanation is that most of the selected participants received statins for secondary prevention of ischemic stroke. Moreover, large prospective studies have demonstrated that statins use is associated with a reduced risk of developing PSE (Guo et al. 2015). Additionally, statins show a dose‐dependent effect. An annual increase of 1 g of atorvastatin is associated with a 5% of reduction in seizure risk, which is possibly due to its anticonvulsant, anti‐inflammatory properties and protective effects on the blood‐brain barrier (Feyissa et al. 2019, Etminan et al. 2010).

In conclusion, this study suggests a bidirectional relationship between epilepsy and ischemic stroke, highlighting the need for further research to clarify the optimal treatment strategies for PSE, including drug interactions and the potential role of prophylactic medication. In the meantime, the possibility of an increased risk of cerebrovascular disease (CVD) after epilepsy should not be ignored (Holtkamp et al. 2017).

Although many elderly patients have complex comorbidities, cerebrovascular disease (CVD) remains the most common comorbidity (Beghi et al. 2023). Patients with LOE have a threefold higher risk of stroke compared to their age‐matched peers without LOE (Johnson et al. 2021). While considering that most patients in this study had ischemic stroke, after excluding cases where stroke was identified as the etiology of epilepsy, the prevalence of stroke as a comorbidity remained consistent with previous studies. In patients with combined ischemic stroke (88.16%), the effectiveness of seizure control was relatively high at 74.25%. A possible explanation is that patients with epilepsy who had CVD who regularly take aspirin had a significantly lower incidence of refractory seizures compared to those without cerebrovascular comorbidities (Alsfouk et al. 2020). In addition, studies have shown that patients with first seizure after the age of 60 years should be considered at high risk of cryptogenic stroke even if no obvious clinical manifestations of CVD. In other words, LOE may serve as a predictive factor for future stroke occurrence. Screening high‐risk individuals for CVD and implementing appropriate interventions could help reduce stroke risk (Brigo et al. 2014, Cleary et al. 2004).

In China, approximately 15.07 million individuals aged 60 and older were living with dementia by 2018, with an overall prevalence of 6.0% (Jia et al. 2020), and at least one‐fourth of patients with newly diagnosed epilepsy occur among those aged 65 and older (Ghosh and Jehi 2014). Consequently, the number of epilepsy patients with comorbid cognitive impairment is expected to increase significantly. However, managing this large patient population is particularly challenging, as individuals with cognitive dysfunction often struggle to accurately report transient loss of consciousness or memory lapses, making diagnosis and treatment more complex. There is a bidirectional association between epilepsy and dementia. Studies have shown that the prevalence of dementia among epilepsy patients ranges from 0.6% to 17.5%, while the incidence of epilepsy in patients with Alzheimer's disease (AD) or other dementias during disease progression is 1.3% to 6.1%, with a significantly higher risk of epilepsy than the general population. According to the Framingham Heart Study (FHS), epilepsy patients have twice the risk of developing dementia compared to the general population. In patients with LOE, the risk of dementia is 2 to 3 times higher than those without LOE (Johnson et al. 2021, Beghi and Beghi 2020, Stefanidou et al. 2020). Our study also found that 10.53% of patients with LOE had comorbid cognitive impairment and dementia, and the effectiveness of seizure control in this group of patients was 46.88%, which was significantly lower compared with patients without dementia.

Studies have shown that the prevalence of comorbidities in patients with epilepsy is significantly higher than in the general population, reaching eight times the rate observed in non‐epileptic individuals, and the prevalence of epilepsy among elderly individuals with psychiatric comorbidities ranges from 8.8 per 1,000 to 29.2 per 1,000 people (Keezer et al. 2016, Martin et al. 2014). A large‐scale study on comorbidities in epilepsy patients reported that 6.2% of epilepsy patients had psychiatric disorders (Giorgia et al. 2021). In our cohort, 24.67% of patients had comorbid psychiatric disorders, which was significantly associated with a poorer 1‐year prognosis, with only 37.33% of patients achieving effective seizure control. An analysis of U.S. Food and Drug Administration (FDA) data on psychiatric medications, extracted from Phase II and III clinical trials involving 75,873 participants, suggested that individuals on regular antidepressant therapy were less likely to experience seizures (Gijsen et al. 2001). Patients with both epilepsy and psychiatric disorders share a common challenge: medication adherence. Clinicians must recognize the rising prevalence of patients with epilepsy having psychiatric disorders, as well as the unique difficulties in diagnosing and treating this population.

This study has the following limitations: 1. The relatively small sample size limits the generalizability of our findings. Larger cohort studies are required to validate these results; 2. As this is a descriptive study, all samples were selected by researchers, which increased the possibility of selection bias; 3. Some patients had a prolonged untreated period before diagnosis. The potential differences in clinical characteristics and prognosis between these patients and those who received immediate medical treatment upon first onset remain unclear; 4. Not all participants underwent MRI examinations, which raises the possibility of neglecting cerebrovascular risk factors; 5. This study primarily evaluated individual risk factors but did not consider the cumulative effects of multiple interacting risk factors, which may have influenced the results; 6. Finally, the population we selected was composed of Chinese individuals, the findings may not be directly applicable to other ethnic groups.

## 5 Conclusions

The primary cause of late‐onset epilepsy (LOE) in elderly patients was ischemic stroke, with focal‐onset seizures being the most common seizure type. Patients with comorbid psychiatric disorders, dementia or cognitive impairment had significantly poorer seizure control within one year.

## Author Contributions

**Jie Hu**: conceptualization, writing–original draft, writing–review and editing, methodology, software, data curation. **Long Wang**: resources, project administration, visualization, writing–review and editing, funding acquisition, investigation, validation, formal analysis. **Jun‐Cang Wu**: funding acquisition, visualization, project administration, resources.

## Conflicts of Interest

The authors declare that they have no known competing financial interests or personal relationships that could have appeared to influence the work reported in this paper.

### Peer Review

The peer review history for this article is available at https://publons.com/publon/10.1002/brb3.70452

## Data Availability

Origin data of this study are available from the corresponding author on reasonable request.
